# Transition from a mixotrophic/heterotrophic protist community during the dark winter to a photoautotrophic spring community in surface waters of Disko Bay, Greenland

**DOI:** 10.3389/fmicb.2024.1407888

**Published:** 2024-06-03

**Authors:** Claudia Sabine Bruhn, Nina Lundholm, Per Juel Hansen, Sylke Wohlrab, Uwe John

**Affiliations:** ^1^Alfred Wegener Institute, Helmholtz Centre for Polar and Marine Research, Bremerhaven, Germany; ^2^Helmholtz-Centre Potsdam, German Research Centre for Geosciences GFZ, Potsdam, Germany; ^3^Natural History Museum of Denmark, University of Copenhagen, Copenhagen, Denmark; ^4^Department of Biology, Marine Biological Station, University of Copenhagen, Helsingør, Denmark; ^5^Helmholtz Institute for Functional Marine Biodiversity at the University of Oldenburg, Oldenburg, Germany

**Keywords:** sea ice, succession patterns, metabarcoding, spring bloom formation, parasites, functional diversity, time series

## Abstract

Unicellular eukaryotic plankton communities (protists) are the major basis of the marine food web. The spring bloom is especially important, because of its high biomass. However, it is poorly described how the protist community composition in Arctic surface waters develops from winter to spring. We show that mixotrophic and parasitic organisms are prominent in the dark winter period. The transition period toward the spring bloom event was characterized by a high relative abundance of mixotrophic dinoflagellates, while centric diatoms and the haptophyte *Phaeocystis pouchetii* dominated the successive phototrophic spring bloom event during the study. The data shows a continuous community shift from winter to spring, and not just a dormant spring community waiting for the right environmental conditions. The spring bloom initiation commenced while sea ice was still scattering and absorbing the sunlight, inhibiting its penetration into the water column. The initial increase in fluorescence was detected relatively deep in the water column at ~55 m depth at the halocline, at which the photosynthetic cells accumulated, while a thick layer of snow and sea ice was still obstructing sunlight penetration of the surface water. This suggests that water column stratification and a complex interplay of abiotic factors eventually promote the spring bloom initiation.

## 1 Introduction

Due to climate change, the Arctic is one of the fastest changing environments in the world (Overpeck et al., 1997; McBean et al., 2005; Rantanen et al., 2022). This has already affected the Arctic biosphere and will lead to further changes in the future (Hoegh-Guldberg and Bruno, 2010). The base of the complex marine pelagic food web consists of unicellular organisms, such as bacteria and eukaryotic unicellular plankton (protists) occupying different ecological niches and providing food for higher trophic levels.

Because of their crucial role in the marine ecosystem, marine protists are frequent study objects. Community studies of Arctic pelagic waters often focus on transect or snapshot studies (Baggesen et al., 2012; Tillmann et al., 2014; Elferink et al., 2017, 2020), which do not properly display the temporal dynamics. To our knowledge, only a limited set of studies investigated seasonal dynamics, focusing on mesopelagic water species (Terrado et al., 2009), or seasonal sea ice and its impact on the protist community (Terrado et al., 2011; Massicotte et al., 2020). Until now, the pelagic winter protist community in the Arctic has been characterized as most likely heterotrophic (Marquardt et al., 2016; Kubiszyn et al., 2017). Some studies indicate that phototrophic diatoms, which are typical spring bloom formers in the Arctic, may survive unfavorable conditions as resting stages (Zhang et al., 1998; McQuoid and Hobson, 2008; Hegseth and Tverberg, 2013; Kvernvik et al., 2018). Other studies have found that diatoms can recover their light harvesting abilities very quickly when it becomes brighter (Lacour et al., 2019), which hints at their activity also during the dark winter period.

The periods with ice cover have been declining during the past decades due climate change. This is expected to impact the timing and dynamics of the spring bloom, and the trophic modes of the protist community (Alexander and Niebauer, 1981; Hunt et al., 2002). Phytoplankton blooms have occasionally been found to develop before the sea ice melts (Arrigo et al., 2012; Spall et al., 2014; Massicotte et al., 2020), and some studies have recognized the abundance of parasitic and mixotrophic protists in the presence of sea-ice (Terrado et al., 2011; Clarke et al., 2019; Søgaard et al., 2021). The seeding of the pelagic phototrophic spring bloom event by sea ice algae has also been considered as an option (Ardyna et al., 2020). While the pattern of phototrophic dominance during the spring bloom event is comparatively well-described (Tammilehto et al., 2017; Lafond et al., 2019; Luostarinen et al., 2020), the community structure of the winter community and its transition toward the vernal bloom is less investigated (Kubiszyn et al., 2017), especially in relation to seasonal sea ice. With this study, we aim to provide insights into the temporal changes in protist community composition from winter to spring, generating a better understanding of the Arctic marine ecosystem. Therefore, we discuss the impact of the occurrence of seasonal sea ice and other abiotic parameters in their interplay with the protist community composition transition from winter to spring in Disko Bay, with special focus on the functional (trophic) groups of the observed organisms.

## 2 Materials and methods

### 2.1 Study site description and sampling procedure

Sampling was performed in Disko Bay off the southern coast of Disko Island, West Greenland, close to the Arctic Station in Qeqertarsuaq. The area is characterized by coastal proximity, annual seasonal sea ice, and influence of the marine terminating Sermeq Kujalleq (Jakobshavn glacier). Samples were taken between February 10 and April 23, 2018, around noon. The sampling started at 69°12.95′ N, 53°31.25′ W, which had a water depth of approx. 140 m. As this location became inaccessible due to sea ice formation and growth, the sampling station was moved to 69°14.2′ N, 53°29.9′ W, depth: *ca.* 140 m, from March 16, 2018, approximately 2.5 km away from the first position. The alternative position was chosen as the best compromise between comparability to the first location and probable accessibility throughout the sampling period. The samples were taken approximately every 4 days with a 25 L Niskin water sampler (KC Denmark, Denmark) either from the water surface or through a manually drilled hole in the ice. The samples, taken at the depths 5 m, 10 m, 20 m, 30 m, and 40 m were transferred to polyethylene containers (pre-treated with 3% hydrochloric acid and flushed twice with the respective sample), stored cold and dark, and processed on the same day.

### 2.2 Sea ice and contextual data

The water sampling was accompanied by an SBE 911plus CTD (Sea-Bird Scientific, Washington, United States) to collect temperature, photosynthetic active radiation (PAR), fluorescence and salinity data. For continuous environmental data above sea level, light from a station located at 69°15′12.558″ N, 53°30′50.863″ W, 25 m above sea level was provided by Greenland Environmental Monitoring (GEM) program, subprogram “GeoBasisDisko.” Sea ice was observed both locally at the sampling location on the sampling day, and daily of the whole bay area by visual sea ice monitoring of the Arctic Station provided by the University of Copenhagen, Denmark.

### 2.3 Sample preparation and analysis

Biomass during the Arctic winter is rather low and because of uniformity with previous studies (Bruhn et al., 2021) we applied a pooling approach of the upper 40 m of the water column. For this, equal volumes (10 L) of water from five depths (5, 10, 20, 30, and 40 m) were pooled to obtain depth-integrated samples of the upper 40 m of the water column. Data for Chlorophyll *a* (Chl *a*), particulate organic carbon and nitrogen (POC and PON) as biomass and nutrition status proxies were retrieved from supplementary material of Bruhn et al. (2021). The remaining 47.5 L pooled sample was size fractionated through serial filtration with multiple filters. Prefiltering through a 200 μm nylon mesh removed most multicellular zooplankton, also resulting in a loss of some larger protist species and colonies. Afterwards, the prefiltered sample was filtered through a 20 μm nylon mesh to obtain the microplankton size fraction (200 μm – 20 μm). Further filtration steps of this filtrate were carried out with polycarbonate filters and a vacuum pump, resulting in the filtration of 3 L through a 3 μm pore size (for obtaining the nanoplankton size fraction, 20 μm – 3 μm) and 1 L through a 0.2 μm pore size (for obtaining the picoplankton size fraction, 3 μm – 0.2 μm). The cells were carefully flushed off the surface of the filters with extraction buffer of a NucleoSpin Soil kit (Macherey-Nagel, Germany). Afterwards, they were frozen at −20°C and transported frozen for extraction in the home institution (AWI). The DNA from these three size fractions (0.2–3 μm or picoplankton, 3–20 μm or nanoplankton, 20–200 μm or microplankton) was extracted using the NucleoSpin Soil kit (Macherey-Nagel, Germany). The 16S rRNA Metagenomic Sequencing Library Preparation protocol (Illumina, California, United States) was used, but adjusted with primers targeting the eukaryotic V4-region (Stoeck et al., 2010) modified to include haptophytes, which are otherwise mostly underrepresented when using the original primers (Piredda et al., 2017). After sequencing 300 bp paired-end with a MiSeq System (Illumina, California, United States), amplicon sequence variants (ASVs) were generated and annotated (as described in Sprong et al., 2020 and with the PR2-database; version 4.11.1). The species were marked with their respective trophic mode, if known, by manual curation (see table in Supplementary data for applied criteria). Afterwards, the 50 most abundant ASVs from each of the taxonomic groups of dinoflagellates, haptophytes, cryptophytes, diatoms and ciliates were determined after excluding low abundance ASVs and non-protist ASVs.

At the next step, ASVs were taxonomically analyzed and their systematic identity confirmed through phylogenetic placement. For this, reference alignments with longer and curated sequences of the different target groups (dinoflagellates, haptophytes, cryptophytes, diatoms or ciliates) have been generated with MAFFT, using the L-INSI settings. The “—add fragments—reorder” option was used to place the ASV fragments into the fixed reference alignment allowing a better precision and likelihood for species assignment. Annotated reference sequences for the alignments were taken from GenBank sequences (NCBI), which were determined by blasting the ASVs from the sequencing run. Afterwards, separate phylogenetic trees for the major taxonomic groups, i.e., dinoflagellates, haptophytes, cryptophytes, diatoms and ciliates, were calculated with RAxML with 1,000 bootstrap analyses, resulting in one maximum likelihood tree per taxonomic group. These trees served as a reference for the phylogenetic assignment or confirmation of the 50 most abundant ASV sequences of the aforementioned taxonomic groups. Alignments and resulting trees have been manually curated and analyzed.

After taxonomic analysis, the protists were grouped into the following functional groups: (1) Photoautotrophs, photosynthetic protists that are not able to take up particulate prey, (2) Heterotrophs, phagotrophic protists (3) Parasites. Mixotrophic organsisms were subdivided in accordance with Mitra et al. (2023), resulting in the functional groups: (1) Constitutive mixotrophs, CMs (i.e., Protists with own chloroplasts, which have the ability to ingest other organisms), (2) Generalist Non-Constitutive mixotrophs, GNCM (i.e., protists without own chloroplasts that can utilize chloroplasts from a wide range of photosynthetic prey), (3) Specialist Non-Constitutive Mixotrophs with endosymbionts, eSNCM (protists without own chloroplasts that can take up whole or reduced symbionts and utilize them), (4) Plastid specialists Non-Constitutive Mixotrophs, protists without own chloroplasts that utilizes only the chloroplasts from specific groups of photosynthetic prey, pSNCM. If the genus was known to contain species of a certain trophic mode, but there was no data available for that particular species, the functional group was marked with a “?”.

Further bioinformatics analyses were performed with R, version 4.0.3 (R Core Team, 2020), with RStudio version 1.3.1093 (R Team) and the packages effects (Fox and Weisberg, 2019), eulerr (Larsson, 2018), ggplot2 (Wickham, 2016), lubridate (Grolemund and Wickham, 2011), MBA (Finley et al., 2017), mgcv (Wood et al., 2016), phyloseq (McMurdie and Holmes, 2013), plyr (Wickham, 2011), RColorBrewer (Neuwirth, 2014), reshape2 (Wickham, 2007), tidyverse (Wickham, 2019), and vegan (Oksanen et al., 2019). Low abundance ASVs and non-protist ASVs were excluded. Read numbers were then normalized to average sequencing depth and afterwards set to 100% reads, to visualize the relative abundance of the ASVs. To facilitate analyses, the samplings were summarized into the three calendar months they were taken in. This resulted in five samplings from February 10 to 27, five samplings from March 7 to 30, and four samplings from April 5 to 23.

## 3 Results

### 3.1 Environmental observations

#### 3.1.1 Oceanographical context

**Figure 1:**
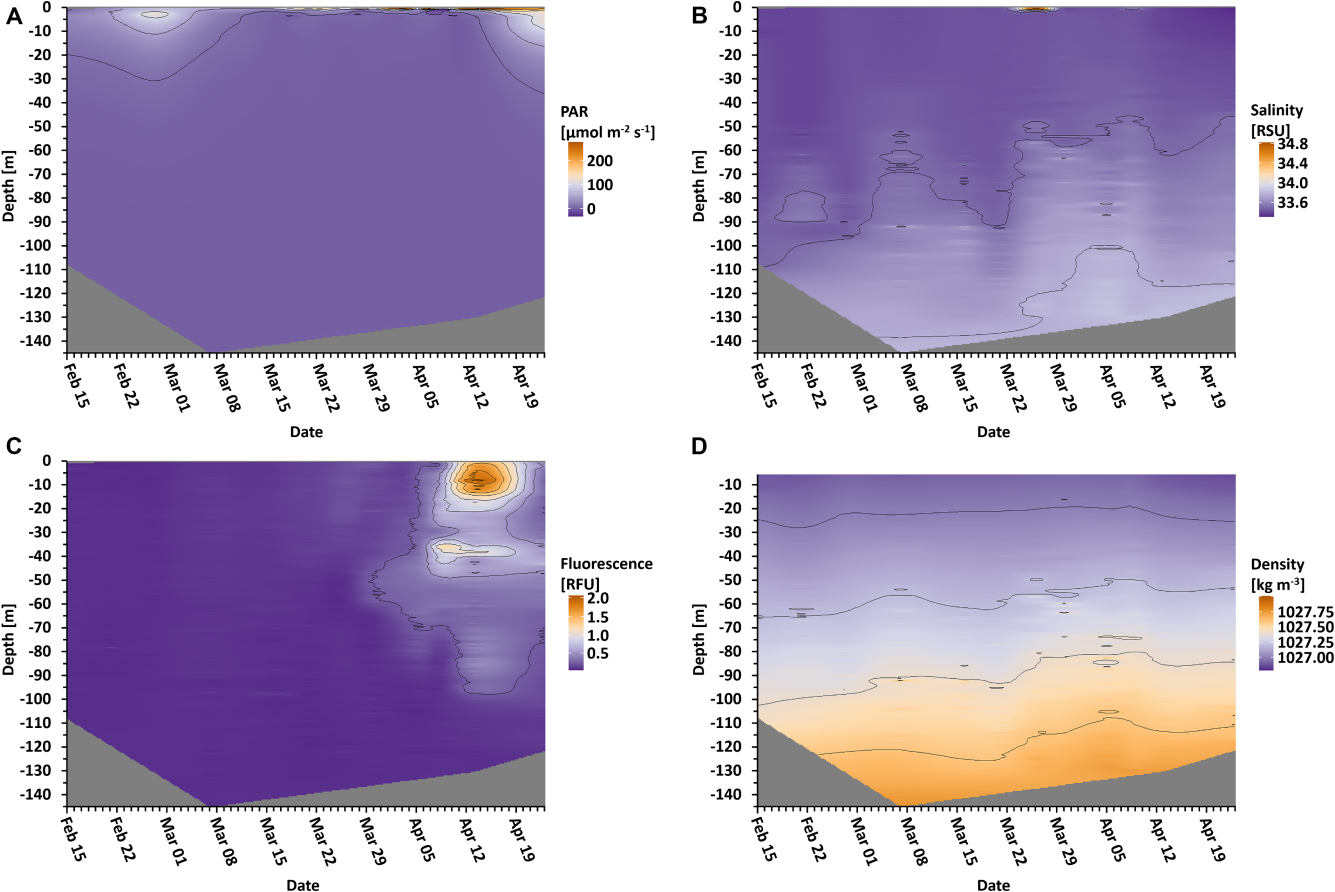
Oceanographic data in depth profile over time. Depicted are photosynthetic active radiation (**A**, PAR), salinity **(B)**, fluorescence **(C)**, and the density of the water **(D)**. Isolines are displayed for orientation regarding the different values. Gray areas indicate unmeasured depths.

The CTD measurements resulted in several depth profiles, of which photosynthetically active radiation (PAR = wavelengths of light within the visible range of 400–700 nm), water density, chlorophyll fluorescence, and salinity are presented (Figure 1). PAR measurements showed some penetration of light into the water at the beginning of the study up until March 7 and again from April 23 and onwards (Figure 1A). Between these dates, there was almost no light penetrating the water column. The measured density of the water column showed a slight shallowing of <20 m of the mixed layer depth (Figure 1D). Fluorescence values started to increase around March 30 at a depth of approximately 55 m (Figure 1C). Additionally, it formed two layers at 40 m and 7 m depth between April 5 and April 9. Afterwards, on April 13, fluorescence was detected as deep as 100 m. Salinity values showed different layers in the water column, which shallowed over time (Figure 1B).

#### 3.1.2 Sea ice presence and light irradiance

**Figure 2:**
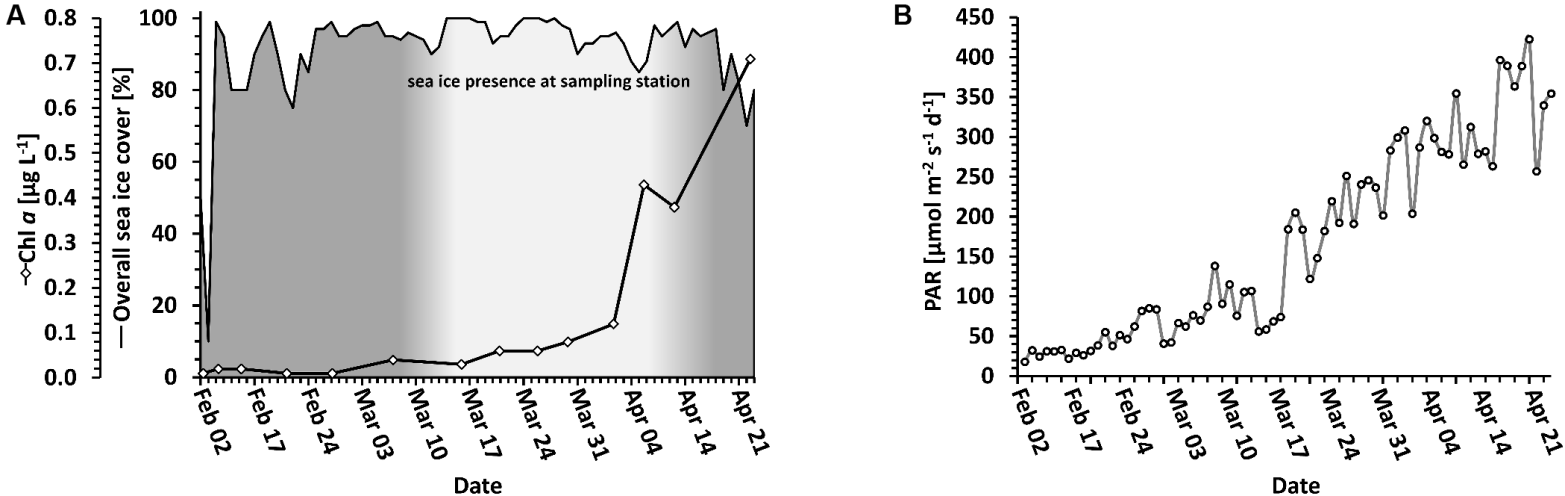
Light and ice conditions. **(A)** Local photosynthetic biomass (solid line with diamonds) in relation to sea ice coverage (solid line). The sea ice coverage of the entire bay area is shown as a black line. The sea ice at the sampling station is indicated as the white coloring below the line. **(B)** Light change over time above water. PAR is displayed as a daily average.

In the following, we distinguish between the overall sea ice presence in the entire Disko Bay area and sea ice directly at the sampling location. Sea ice was present but did not cover the full bay throughout the whole period. In the Disko Bay area, the sea ice cover reached a maximum coverage of 99% on February 12, and covered at least 75% until April 25, when the ice slowly started to break up (Figure 2A, black line). At the sampling station, sea ice was building up between March 7 and March 16 (Figure 2A, white area), when it reached a thickness of more than 40 cm with an additional snow cover. After April 5, the ice at the sampling station began to melt again, rendering the sampling on April 13 to be from the sea ice edge and the sampling on April 23 from the water surface. The day length above the water naturally increased during the sampling period, which therefore led to an increased daily dosage of PAR (Figure 2B), of which only a fraction penetrated the upper ocean layers (Figure 1A).

### 3.2 Protist biomass and community structure changes

Particulate organic carbon (POC), particulate organic nitrogen (PON) and chlorophyll *a* (Chl *a*) were used as proxies for biomass. POC and PON were measured to 63.7 μg mL-1 POC and 4.9 μg L-1 PON on the first day of measurement (February 10) and decreased until 14.0 μg L-1 POC on March 21 and 0.8 μg L-1 PON on February 21 (Table 1). Afterwards, both POC and PON increased until the end of the sampling campaign to their highest values of 70.8 μg L-1 POC (on April 23) and 12.7 μg L-1 PON (on April 13). In contrast, Chl *a* gradually increased from 0.01 μg L-1 on February 21 to 1.26 μg L-1 on April 19 (Figure 2A). The curve follows an exponential function with an *R*^2^ = 0.9224.

**Table 1:** POC, PON, and Chl *a* as biomass proxies.

	Feb 10	Feb 15	Feb 21	Feb 27	Mar 07	Mar 16	Mar 21	Mar 26	Apr 05	Apr 13	Apr 19	Apr 23
POC [μg L^−1^]	68.40	63.72	43.29	16.36	33.46	31.41	14.01	16.18	23.79	66.13	49.82	70.79
PON [μg L^−1^]	3.73	4.94	0.8	6.44	6.41	5.83	3.77	3.51	4.64	12.67	8.24	12.19
Chl a [μg L^−1^]	0.01	0.02	0.01	0.01	0.04	0.03	0.06	0.06	0.12	0.38	*NA*	0.71
POC:PON	18.31	12.90	54.25	2.54	5.22	5.39	3.71	4.61	5.13	5.22	6.05	5.81

Data were retrieved from Bruhn et al. (2021).

**Figure 3:**
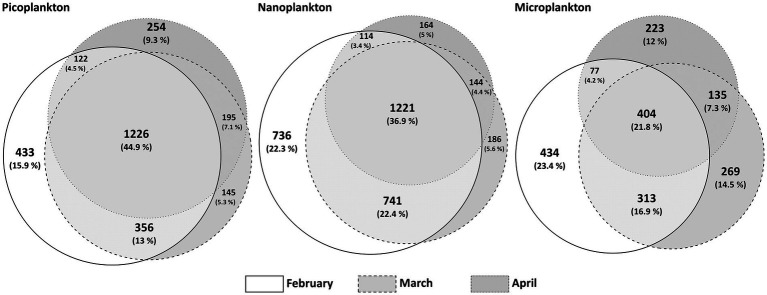
Venn-Diagram adaptation of ASVs per month and size fraction. A presence/absence-matrix was the basis for this visualization, where shared ASVs per calendar month are depicted in the overlaps. The circles are proportional to the number of unique ASVs.

In total, 4,009 different ASVs were assigned to protists in the metabarcoding analyses. The 300 most abundant protist ASVs accounted for 81 to 98% of all reads, depending on the sampling date, of which 97% were present in all 3 months, albeit with strongly varying relative read abundances of the different groups and taxa. ASVs that were unique to a certain month were the overall least abundant ASVs, ranging from 14.3% (February exclusive ASVs) over 5.4% (April exclusive ASVs) to 4.7% (March exclusive ASVs) of all sequence reads assigned to ASVs. A range from 44.9% in picoplankton, over 36.9% in nanoplankton to 21.8% in microplankton of all protist ASVs were shared among all 3 months (Figure 3). The highest number of unique ASVs per month is detected in February and the smallest number in April.

**Figure 4:**
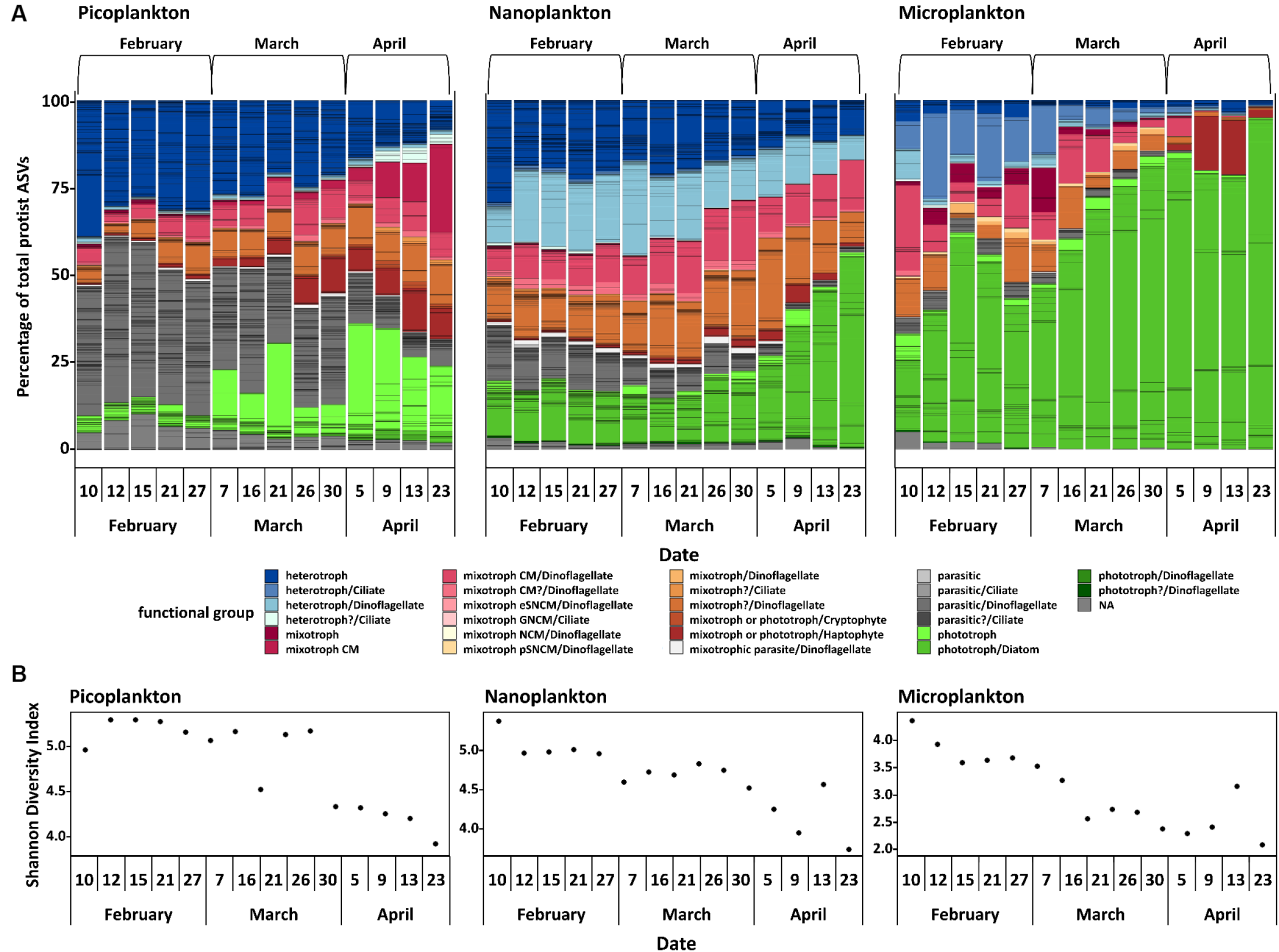
Protist community analyses. Normalized protist ASVs, divided by functional group and size fraction and additionally divided into three calendar months **(A)**. CM, constitutive mixotroph; eSNCM, endo-symbiotic specialist non-constitutive mixotrophs; GNCM, generalist non-constitutive mixotrophs; NCM, non-constitutive mixotroph; pSNCM, plastidic specialist non-constitutive mixotrophs. It was not possible to assign the definite trophic mode to each ASV, hence a putative trophic mode (indicated with a question mark or NA) is displayed. The Shannon Diversity Index **(B)** is also displayed.

In February, the protist communities in all size fractions were mostly heterotrophic, parasitic and mixotrophic. The percentage of ASVs linked to heterotrophic taxa declined strongly during the sampling period, whereas ASVs linked to phototrophic taxa increased with time leading to a phototroph dominated community in April (Figure 4A). ASVs linked to phototrophic taxa were mainly diatoms, especially in the nanoplankton and microplankton size fractions. In February, considerable percentages of picoplankton and nanoplankton reads accounted for parasitic protists. In March and April, this functional group was continuously displaced by mixotrophs. Over time, Shannon diversity declined in all size fractions (Figure 4B). Picoplankton and nanoplankton have significantly different Shannon diversity indices between the 3 months [with ANOVA, *F*(2, 12) = 33.1, *p* < 0.05 for picoplankton and *F*(2, 12) = 16.6, *p* < 0.05 for nanoplankton], with significantly lower Shannon diversity indices in April compared to February and March, but no difference between February and March (Tukey adjusted *p*-values < 0.05). In the microplankton fraction, the 3 months also differed significantly [ANOVA, *F*(2, 12) = 16.4, *p* < 0.05], with significantly lower Shannon diversity indices in April and March compared to February, but no difference between April and March (Tukey adjusted *p*-values < 0.05).

**Figure 5:**
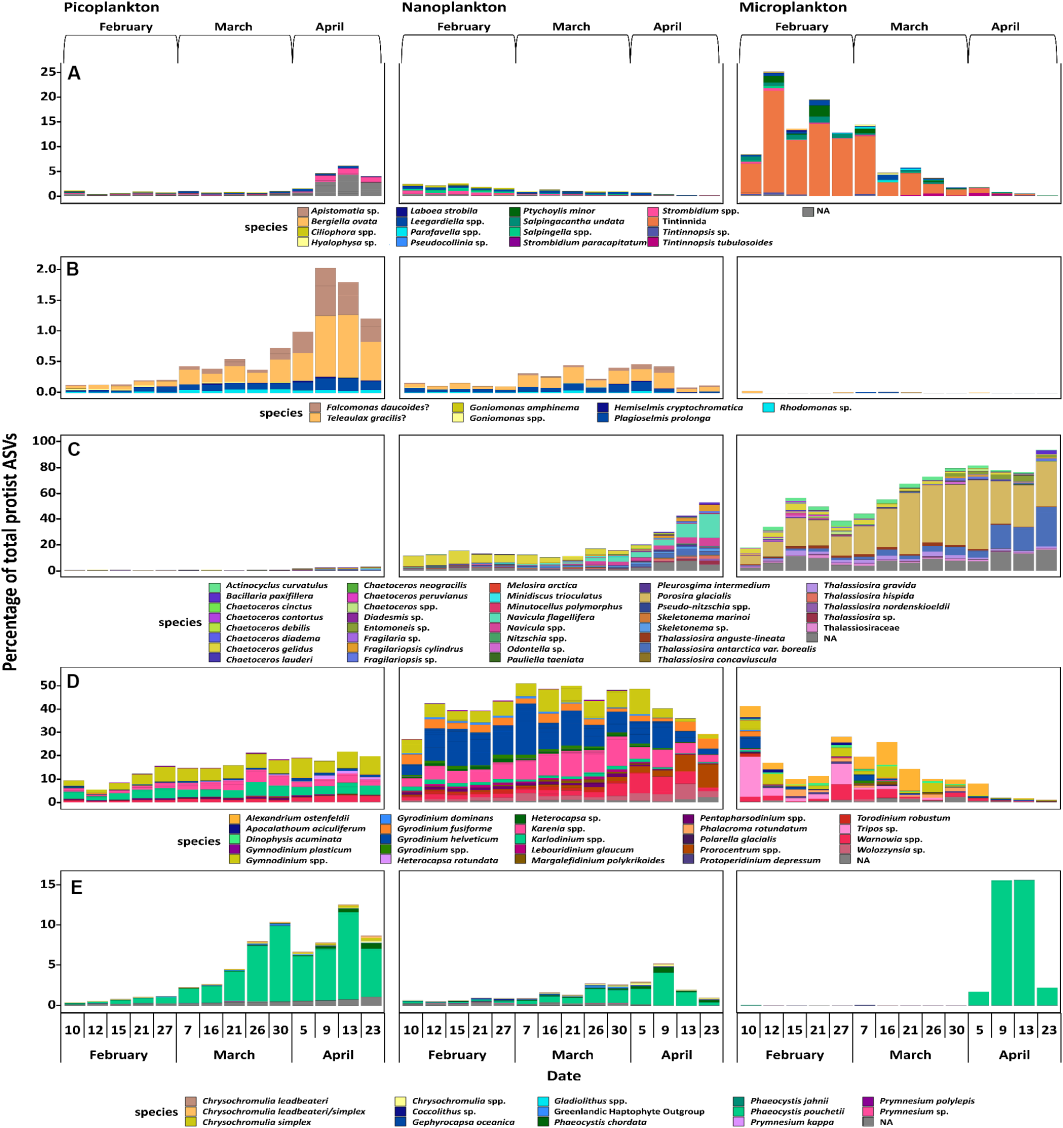
most important ASVs of the taxonomic groups of ciliates **(A)**, cryptophytes **(B)**, diatoms **(C)**, dinoflagellates, excluding Syndiniales **(D)**, and haptophytes **(E)**. Displayed are a maximum of the 50 most abundant ASVs of each taxonomic group, if applicable. Each species name is to be understood as putative, as the species themselves were not confirmed via microscopic investigation but only through phylogenetic placement.

When evaluating the 50 most abundant ASVs of ciliates, cryptophytes, diatoms, dinoflagellates (excluding Syndiniales), and haptophytes individually, the successional patterns of some putative species stand out (Figure 5). In the following, the putative species belonging to the ASVs will be called by the respective species name assigned after phylogenetic placement analyses and are meant as presumed (phylo)species names. Ciliates were diverse and difficult to identify to species level. Most noteworthy, one ASV of an unidentified heterotrophic tintinnid declined in abundance in the microplankton size fraction, accounting for >20% of all microplankton reads on February 12 to <2% on April 23 (Figure 5A). Cryptophytes, which are either mixotrophs or phototrophs, were mainly found in the picoplankton size fraction. Here, *Teleaulax gracilis*, *Falcomonas daucoides* and the *Plagioselmis* stage of *Teleaulax amphioxeia* all increased in abundance with time (Figure 5B). The most abundant diatom in the microplankton size fraction was *Porosira glacialis*, followed by *Thalassiosira antarctica* var. *borealis*. In the nanoplankton, the most abundant diatoms were *Chaetoceros gelidus*, *Navicula flagillifera* and other *Navicula* species. *Chaetoceros gelidus* had the highest relative abundance in February and March, declining with time. On the other hand, *Navicula flagellifera* and other *Navicula* spp. were the most relatively abundant diatoms toward the bloom initiation in April. *Skeletonema* sp. was the most abundant diatom of the picoplankton size fraction, and it increased in relative abundance during bloom initiation in April (Figure 5C).

Overall, dinoflagellates made up the most abundant group based on absolute sequence read numbers. Metabarcoding approaches targeting the rRNA gene tend to overestimate the abundance of dinoflagellates, because their genome usually displays a high copy number of ribosomal operons (Guo et al., 2016), making a direct comparison across groups challenging. However, this is less impacted when comparing within a group. All of the 50 most abundant dinoflagellate taxa are most likely heterotrophs and constitutive mixotrophs (CMs). A constitutive mixotroph has a constitutive ability to conduct photosynthesis and phagocytise (Flynn et al., 2019). In the picoplankton size fraction *Gymnodinium* spp. and *Karenia* sp. increased in relative abundance over time, whereas *Karlodinium* sp. stayed at the same relative level throughout the study period. In the nanoplankton, *Gymnodinium* spp. neither increased nor decreased, while *Tripos* sp. and *Prorocentrum* sp. increased in the spring period, whereas *Karenia* sp. and *Gyrodinium* sp. decreased. In the microplankton size fraction, *Torodinium robustum* and Tripos sp. decreased in relative abundance. *Alexandrium ostenfeldii* was also a fairly abundant species in the microplankton size fraction, and was present throughout the whole sampling period, but had a very low relative abundance from April 9 on (Figure 5D).

When analyzing haptophytes, a clade of six unidentifiable ASVs was found, which were distantly related to *Chrysochromulina* spp. The mixotroph/phototroph *Phaeocystis pouchetii* was the most prominent haptophyte. It increased in relative abundance over time in all three size fractions, existing both as single cells and in large colonies. In the microplankton fraction, *P. pouchettii* was almost non-existent until April 9, whereas in the nanoplankton fraction, it gradually increased in abundance and peaked on April 9 (Figure 5E).

## 4 Discussion

The method of size fractionation might have impacted the integrity of more fragile cells, which could have fragmentized under the pressure of the vacuum filtration. The reads of a very few fragile larger taxa such as *Strombidium* spp. (Figure 4A) in the picoplankton size fraction may have been the result of this method. On the other hand, these findings could also hint at the presence of considerably smaller gametes. Additionally, this method allows, e.g., for insights in seasonal colony formation of the haptophyte *Phaeocystis pouchetii*, and was successfully applied in other field studies several times (Krock et al., 2009; Elferink et al., 2020; Bruhn et al., 2021), which was the reason why it was used for this study.

The winter communities were dominated by parasites, heterotrophs and mixotrophs during February (Figure 4A). In more temperate coastal regions, where more light is available, small heterotrophic protists were also dominating the winter population (Morán et al., 2018). However, especially the picoplankton and nanoplankton size fractions revealed a high relative abundance of parasitic organisms during winter, and not only general heterotrophs. At times, the picoplankton fraction consisted almost entirely of parasites and heterotrophs, which underlines the importance of these two trophic modes for the winter community. Most marine parasitic protists are relatively small and target considerably larger cells as host organisms (Gómez et al., 2009; Alacid et al., 2015; John et al., 2019), indicating that most of the parasitic protists detected in the study were probably in their free-living stage, showing up in the picoplankton fraction. Very few parasites were detected in the microplankton fraction, further supporting the conjecture that few of the parasites were inside microplankton host cells, unless these cells were broken up by, or external parasites that have been dislodged during the filtration process. On the other hand, the low abundance of reads assigned to parasites in the microplankton size fraction could also be accounted for by a higher DNA content of larger cells, which in turn could dominate over the comparably low relative abundance of endoparasites in the PCR-based method. In Antarctic waters, parasitic protists have been detected as being surprisingly prevalent in winter (Cleary and Durbin, 2016), probably associated with the sea ice lead, i.e., long openings in the sea ice cover (Clarke et al., 2019). Most of the parasitic organisms were dinoflagellates, specifically Syndiniales. These often infect ciliates, dinoflagellates, cercozoons, and crustaceans (Guillou et al., 2008), i.e., groups of mixotrophic and heterotrophic organisms, but apparently not or only rarely diatoms (Tillmann et al., 1999). In Disko Bay, heterotrophic and especially mixotrophic dinoflagellates were detected in all size fractions. The overall biomass (assessed as POC) was, however, extremely low (Table 1). Little is known about the autecology of parasitic dinoflagellates in the ocean, in particular because they are difficult to maintain in culture. Parasitic protists usually do not stay alive for prolonged periods of time without their host organisms, and they typically complete their free-living stages within a few hours to days (Alacid et al., 2015; Reñé et al., 2017; John et al., 2019). The existing laboratory experiments suggest that they are not fit to live without their host organisms for an extended period of time (Alacid et al., 2015; John et al., 2019). Resting spores as an overwintering strategy for parasites have not been described yet, although such a strategy is a possibility (Guillou et al., 2008). It is possible that the parasitic organisms observed were simply very successful in finding their host organisms and completing their life cycles with an output of many new individual cells (dinospores), but we cannot exclude alternative survival strategies.

The presence of mixotrophic organisms, mainly CMs, may be related to them having had an advantage over organisms which are less flexible in their trophic mode, because they gain energy from both harvesting the little light available and additional food uptake.

Also later, during the early stages of the spring bloom, mixotrophs, especially CM dinoflagellates, contributed substantially to the total photosynthetic protist community in the pico- and nanoplankton size fractions (March). This may have been a response to the slightly increased day length (Figure 2B), although the light reaching into the water was still negligible (Figure 1A). Similar observations in the community structure have been made in the Young Sound fjord in Northeast Greenland. Here, a bloom of mixotrophic haptophytes developed in ice covered surface waters during early spring (Søgaard et al., 2021). The two locations differ considerably with regard to salinity and nutrient concentrations. Nevertheless, mixotrophs seemed to have had an advantage at both locations, because they compensate for low levels of photosynthesis with their ability to ingest other organisms. The mixotrophic ability seems to give them the flexibility to quickly adapt to increasing light availability, thereby giving them an advantage over pure photoautotrophs at this seasonal time point. It is even possible that mixotrophy dominates the pelagic food web during much of the year in the Arctic, due to this increased persistence (Stoecker and Lavrentyev, 2018).

Chlorophyll *a* concentration followed an exponential growth curve throughout the sampling period (Figure 2A). Additionally, an increase of phototrophs in relative abundance in all size fractions can be seen (Figure 4A). These two changes over time showed the spring bloom initiation. A large relative abundance of photosynthetic diatoms, especially in the nanoplankton and microplankton size fractions, mainly characterized the spring bloom community in April. In the dark winter period in the Arctic, light as the primary source of energy for phototrophs is naturally lacking, while inorganic nutrients are sufficient. One known possible overwintering strategy for diatoms are resting spores, which can germinate when the conditions are more favorable (McQuoid and Hobson, 1995; Tsukazaki et al., 2019; Luostarinen et al., 2020). Another strategy for fast adaptation to better conditions of phototrophs, mainly diatoms, is the quick photosynthetic reactivation of resting cells after a period of darkness (Kvernvik et al., 2018), as dormant cells only display a much-reduced photosynthetic capacity. The presence of diatoms throughout all months, although at times in small proportions, suggests the utilization of the latter or both strategies. As stated before, diatoms are usually not the primary target of the parasitic Syndiniales. Diatoms seem to combine the advantages of the ability to photosynthesize and efficient nutrient uptake. Being r-strategists, surviving as resting cells and with not being targeted prominently by parasitic Syndiniales organisms, they might be avoiding much biotic pressure at the start, thus possibly giving them the critical advantage for overgrowing the other organisms both proportionally and in absolute abundance, leading to the spring bloom event.

Diatoms are typical spring bloom organisms and are often the dominant group in Arctic spring blooms (Tammilehto et al., 2017; Krause et al., 2018; Lafond et al., 2019; Bruhn et al., 2021). The genera *Thalassiosira* spp. and *Navicula* spp. Have previously been detected as important spring bloom species in the Baffin Bay area, not far from the sampled position, although much later in the year and two years prior in 2016 (Lafond et al., 2019). *Porosira glacialis* is also a cold-water diatom, commonly found in the northern hemisphere (McMinn et al., 2005; Svenning et al., 2019), and it was also one of the dominating phototrophs in the microplankton size fraction (Figure 5C).

The haptophyte *Phaeocystis* spp., generally *P. pouchetii*, is an important Arctic phytoplankton species especially toward the late spring bloom and summer (Marquardt et al., 2016), but can also be found in Arctic waters during the entire winter (Vader et al., 2015; Marquardt et al., 2016). On occasion, they have been detected in under-ice blooms as well (Pavlov et al., 2017; Ardyna et al., 2020). *Phaeocystis* spp. are considered a less desirable food source for zooplankton compared to other phytoplankton taxa (Weisse et al., 1994; Nejstgaard et al., 2007). In the presented study, *Phaeocystis pouchetii* started as solitary cells in February and March (in the picoplankton fraction) making them potential prey for microplankton (Figure 5E). This is in accordance with findings, e.g., in Svalbard (Hegseth et al., 2019), where *Phaeocystis* spp. also was initially present as single cells and progressing to colonies later on. Later in April, toward the bloom, it started to form larger colonies. This is similar to Wassmann et al. (2005) where *P. pouchetii* started forming colonies during sea ice retreat. The colony formation observed here may have been a defense mechanism against smaller copepod species (Nejstgaard et al., 2007). While larger copepods, such as *Calanus* spp., are able to graze on these colonies, *Phaeocystis* spp. do not appear to make up a significant part of *Calanus* spp. diet (Ray et al., 2016). *Phaeocystis* spp. have an advantage over diatoms because they are not dependent on silicate concentrations, which diminish quickly during the spring bloom (Bruhn et al., 2021). Compared to some other Arctic phytoplankton species, *Phaeocystis* spp. have a wider tolerance toward temperature, as they are also commonly found in the Atlantic (Hoppe et al., 2018), but may only be able to compete against temperate species to a limited degree (Ahme et al., 2023). This increased fitness makes them a possible candidate for gaining importance in the Arctic spring bloom event in the future. We can confirm the presence of *P. pouchetii* in the winter community in Disko Bay, as also shown close to Svalbard (Vader et al., 2015), underlining the far distribution of this predominantly Arctic species.

The diversity analyses based on metabarcoding and resulting ASVs showed that the community in winter was generally more diverse than toward and during the spring bloom event (Figure 4B). Interestingly, the smaller the organisms, the more similar the communities of the different months were in terms of presence or absence of ASVs (Figure 3). The largest community differences were thus seen in the microplankton size fraction, in which only 21.8% of ASVs were shared among all sampling months. These findings are similar to a comparative study of ASVs from Iceland and Greenland (Elferink et al., 2020), in which the microplankton size fraction was most dissimilar in the different regions compared to smaller size fractions. These findings are related to plasticity and their boundaries of species and ecotype forming (Wohlrab et al., 2018; Elferink et al., 2020). Smaller species have smaller genomes and higher generation times and therefore might evolve faster and differentiate more rapidly into distinct ecotypes, i.e., different ecotypes but same species. Hence, the same species can progress over the seasonal transition, as in this study we do have more species but less changes over time providing a different strategy of some adaptational flexibility (Wohlrab et al., 2018; Elferink et al., 2020). Ecotype forming within species complexes have been well documented, e.g., for prokaryotes as *Synechococcus* and *Prochlorococcus* (Sohm et al., 2015). Genome size and its inverse correlation to fitness has been documented, e.g., for the eukaryotic alga *Dunaliella tertiolecta* (Malerba et al., 2020). In addition, parasites have been documented to reduce their genomes by gene loss and elimination of any secondary DNA to achieve higher fitness in response to their hosts (as also reviewed by Thomas Cavalier-Smith, 2005).

In a global context, it has been shown that the highest phytoplankton diversity is often detected at intermediate biomasses, while especially high and low biomass correlate with lower diversity (Irigoien et al., 2004). In our case, we found that the low biomass winter community was surprisingly diverse (Figure 3B), and that the diversity, by means of ASVs and Shannon diversity index, decreased with the onset of the spring bloom. This suggests a highly diverse winter community followed by a spring bloom, in which fewer species and mostly diatom ASVs started to dominate the community in both relative and absolute abundance, as the conditions became favorable for them. Additionally, as described above, the overall less diverse microplankton size fraction show larger changes by means of community shifts to a changing environmental conditions over the sampling period than the smaller size fractions, supporting the hypothesis that larger celled species have a larger niche space, but cannot rapidly adapt to the seasonal changes due to their reaction norm limits. In temperate regions, food-web shifts are also discussed as a possible factor for plankton bloom initiations (Behrenfeld and Boss, 2014), which could be supported by the trophic shifts in the community of this study for this Arctic environment as well.

Studies in the Arctic have been investigating the phytoplankton spring bloom both in areas with sea ice (Terrado et al., 2008; Massicotte et al., 2020) and without sea ice (Kubiszyn et al., 2017). The ice cover has often been discussed as a factor involved in the initiation of the spring bloom because snow and ice cover will lower the penetration of light into the water column, depriving phototrophs of their energy source (Terrado et al., 2008; Leu et al., 2015). However, the transition from a sea ice covered surface water environment to surface waters without sea ice cover has rarely been studied. Here, we present data on the bloom dynamics starting in the dark winter period to the sea ice break-up and formation of a spring bloom. The slow increase in Chl *a* shows the initiation of the spring bloom event at a time when the sea ice was still largely covering the Bay (Figure 2A, Table 1). Biomass is, at this time, not yet strongly increasing, but when taking POC into consideration, the amount of phototrophs (measured as Chl *a*, Table 1) is starting to dominate the total amount of biomass, showing the imminence of the spring bloom.

Several studies have shown that phytoplankton growth is possible under very low light conditions, as often observed in surface waters under sea ice (Arrigo et al., 2012; Spall et al., 2014; Massicotte et al., 2020). It has also been shown that once light is available after the dark season, photosynthetic capabilities are quickly reactivated, usually within a few hours to a day (Kvernvik et al., 2018; Lacour et al., 2019). In the present study, the photon flux per day above water changed considerably (Figure 2B), but the light penetrating the ice was still extremely limited at the time of increasing photosynthetic activity (Figures 1A,C). The fluorescence measurement shows that photosynthetic cells were first gathering at a depth of approximately 55 m, which coincided with the approximate halocline at that time (Figures 1B,C), and which contrasts with previous findings of photosynthetic cells higher in the water column (Rat’kova and Wassmann, 2002). It is likely that the photosynthetic cells accumulated at this layer, e.g., due to increased density beyond this depth, marking the lower border of the ocean’s mixed layer. The decrease of this mixed layer depth over time (Figures 1B,D) could have possibly led to the cells being able to stay closer to the surface for a longer amount of time to be able to harvest sufficient photons for growth. Our study suggests that the pelagic spring bloom was not seeded from the sea ice or from the bottom of the sea ice as pennate diatoms typically dominate sea ice communities. Instead, we observed typical centric pelagic bloom species, similar to the findings of Arrigo et al. (2008, 2017). This suggests the seeding of the bloom from algal cells that were already present in the water column (as considered by Ardyna et al., 2020), but accumulated at the mixed layer depth. It is possible that the ongoing sea ice break-up in the vicinity could have additionally led to increased turbulences in the upper ocean layers. This could support non-motile cells such as diatoms to stay in the illuminated layers of the ocean, increasing the amount of possibly absorbed photons due to residence in lighter areas of the ocean, eventually enabling their growth. During the initiation of the spring bloom, the local area was still completely covered with sea ice. However, open patches further away from the sampling area may have been sufficient to increase the mixing in the suggested way and to lead to advective effects.

## 5 Conclusion

During winter, the low biomass but highly diverse protist community mostly consisted of parasites, heterotrophs, and mixotrophs, which is probably a natural adaptation to a life at low light availability (Clarke et al., 2019; Søgaard et al., 2021). The transitional period was characterized by a high relative abundance of mixotrophs, which most likely have a trophic advantage due to their trophic flexibility. The community shift toward a spring bloom community already started before the sea ice retreated. Past studies have forecasted and shown an increase in primary productivity after sea ice retreat, based on satellite data (Arrigo et al., 2008; Renaut et al., 2018). However, *in situ* studies, such as ours, confirm that blooms of microbial plankton not only occur (Arrigo et al., 2012; Spall et al., 2014; Pavlov et al., 2017; Massicotte et al., 2020; Søgaard et al., 2021), but also start growing while ice is still covering the surface waters. We also show that the period prior to the phytoplankton spring bloom is most likely not a period of dormancy, but that changes in the low biomass community are occurring (as also observed by, e.g., Marquardt et al., 2016). This suggests that sea ice retreat may not be the major factor in initiating the phytoplankton spring bloom in the Arctic. Rather, an interplay of light intensity, spectral composition and day-length, as well as oceanographic factors such as nutrient availability and halocline depth, i.e., mixed layer depth, are involved, making the spring bloom initiation and the shift from the winter community a multifactorial event (as also suggested by Ardyna et al., 2020).

## Data availability statement

The datasets presented in this study can be found in online repositories. The names of the repository/repositories and accession number(s) can be found in the article/Supplementary material.

## Author contributions

CB: Writing – review & editing, Writing – original draft, Investigation, Formal analysis, Data curation. NL: Writing – review & editing, Writing – original draft, Supervision. PH: Writing – review & editing, Writing – original draft, Methodology. SW: Writing – review & editing, Writing – original draft, Methodology, Formal analysis. UJ: Writing – review & editing, Writing – original draft, Supervision, Resources, Project administration, Funding acquisition.
