# Supplementary material for: Transition from a mixotrophic/heterotrophic protist community during the dark winter to a photoautotrophic spring community in surface waters of Disko Bay, Greenland
Source: Front Microbiol. 2024 Jun 3;15:1407888. doi: 10.3389/fmicb.2024.1407888 (PMC11180815; doi:10.3389/fmicb.2024.1407888)
Supplement: Supplementary file 1 [file Table_1.DOCX]

**Supplementary Table 1:** Criteria for assigned trophic mode of organism.

| Assigned trophic mode | Assigned to |
| --- | --- |
| heterotroph | Anything not within the other heterotroph groups that lacks photosynthetic plastids |
| heterotroph/Ciliate | A phagotrophic (heterotrophic) ciliate, that does not follow a parasitic lifestyle |
| heterotroph/Dinoflagellate | A heterotrophic dinoflagellate – a phagotrophic dinoflagellate without chloroplasts that does not display a parasitic lifestyle |
| heterotroph?/Ciliate | A ciliate that is suspected to be a phagotrophic heterotroph, and does not display a parasitic lifestyle |
| mixotroph | Mixoplankton – a planktonic protist that combine *phototrophy* and *phagotrophy* |
| mixotroph CM | A constitutive mixoplanktonic protist that has an innate, constitutive, ability to conduct photosynthesis and that is also able to phagocytise. (Cf. *NCM*.) |
| mixotroph CM/Dinoflagellate | A dinoflagellate with its own chloroplast(s) that combines phototrophy and phagotrophy |
| mixotroph CM?/Dinoflagellate | A dinoflagellate that is suspected being a constitutive mixotroph |
| mixotroph eSNCM/Dinoflagellate | A specialist non-constitutive mixoplanktonic dinoflagellate that harbours endo/ecto symbionts |
| mixotroph GNCM/Ciliate | A generalist non-constitutive mixoplanktonic ciliate that lacks an innate, constitutive, ability to perform photosynthesis and acquires its phototrophic potential from various other organisms. (Cf. CM, GNCM, SNCM.) |
| mixotroph NCM/Dinoflagellate | A non-constitutive mixoplanktonic dinoflagellate that lacks an innate, constitutive, ability to conduct photosynthesis and thus acquires its phototrophic potential from (an)other organism(s). (Cf. CM, GNCM, SNCM.) |
| mixotroph pSNCM/Dinoflagellate | A plastidic specialist non-constitutive mixoplankton; these acquire and exploit only the plastids originating from another organism. (Cf. CM, GNCM, SNCM.)  Q10: |
| mixotroph/Dinoflagellate | Mixoplanktonic dinoflagellate |
| mixotroph?/Ciliate | A ciliate suspected of a mixotrophic lifestyle |
| mixotroph?/Dinoflagellate | A dinoflagellate suspected of a mixotrophic lifestyle |
| mixotroph or phototroph/Cryptophyte | A photosynthetic cryptophyte in which it is unknown to which extent is capable of phagotropy |
| mixotroph or phototroph/Haptophyte | A photosynthetic haptophyte in which it is unknown to which extent is capable of phagotropy |
| mixotrophic parasite/Dinoflagellate | A parasitic dinoflagellate which has its own chloroplasts |
| parasitic | The organisms are classified as parasitic, but do not fit into the other categories |
| parasitic/Ciliate | A parasitic ciliate |
| parasitic/Dinoflagellate | A parasitic dinoflagellate, in essence belonging to Syndiniales |
| parasitic?/Ciliate | A ciliate that is suspected of being a parasite |
| phototroph | A photosynthetic organism |
| phototroph/Diatom | Diatoms are exclusively phototrophic – they do not have the ability of phagocytosis; therefore all diatoms were summarized in this category |
| phototroph/Dinoflagellate | A phototrophic dinoflagellate |
| phototroph?/Dinoflagellate | A dinoflagellate that is suspected of being a phototroph |
| NA | It was not possible to assign a trophic mode, either due to poor identification or to lack of information on the assigned taxon |
