# Supplementary figures and images for: Transition from a mixotrophic/heterotrophic protist community during the dark winter to a photoautotrophic spring community in surface waters of Disko Bay, Greenland
Source: Front Microbiol. 2024 Jun 3;15:1407888. doi: 10.3389/fmicb.2024.1407888 (PMC11180815; doi:10.3389/fmicb.2024.1407888)

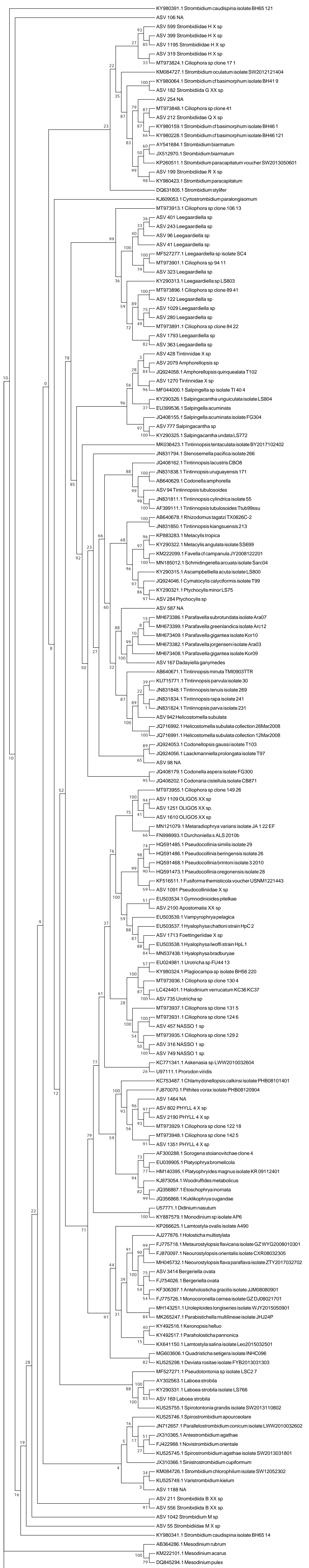

Supplement: Supplementary file 4 [file Data_Sheet_1.PDF]

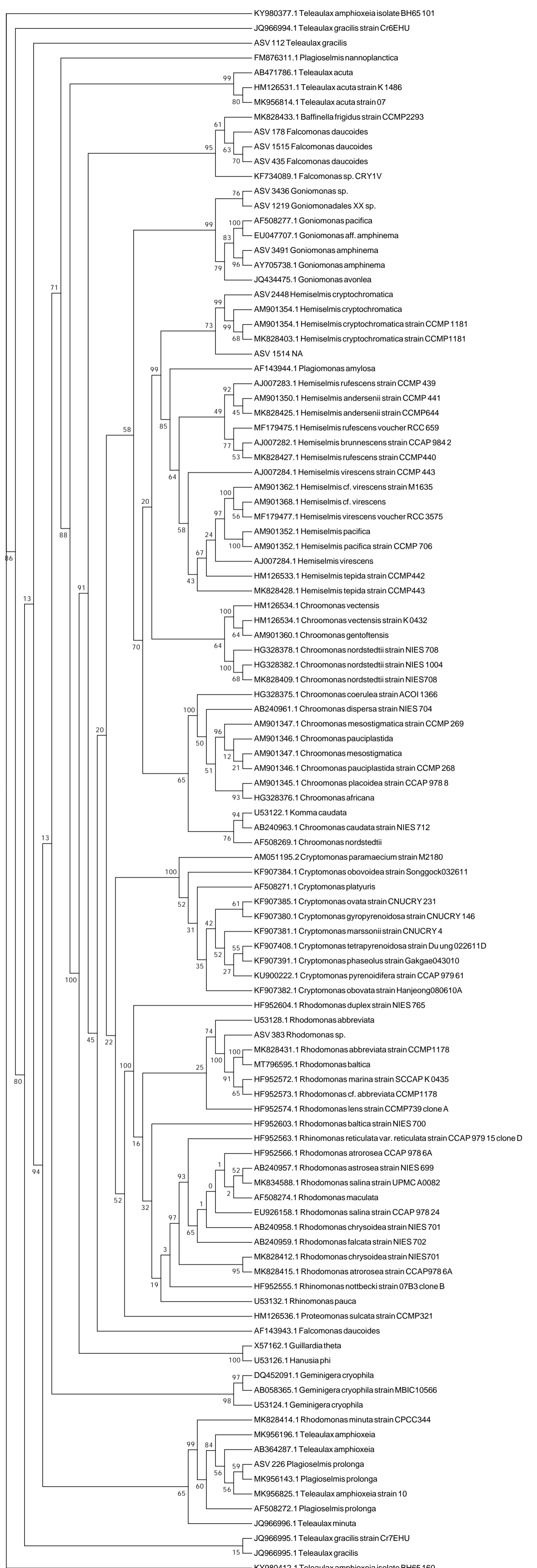

Supplement: Supplementary file 5 [file Data_Sheet_2.PDF]

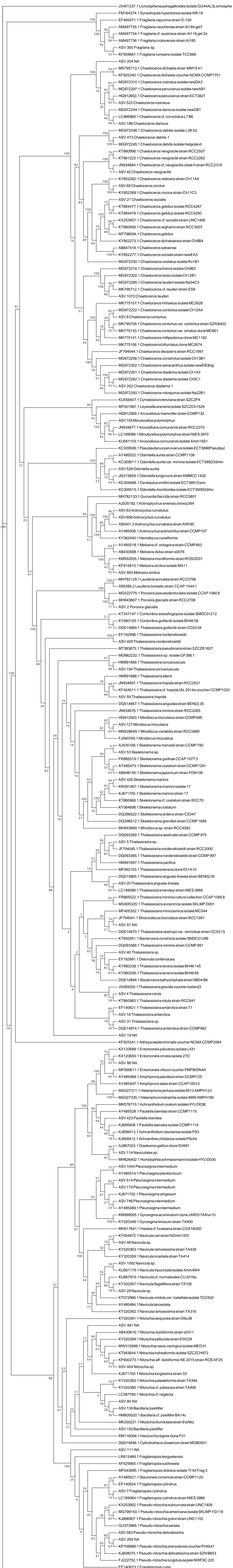

Supplement: Supplementary file 6 [file Data_Sheet_3.PDF]

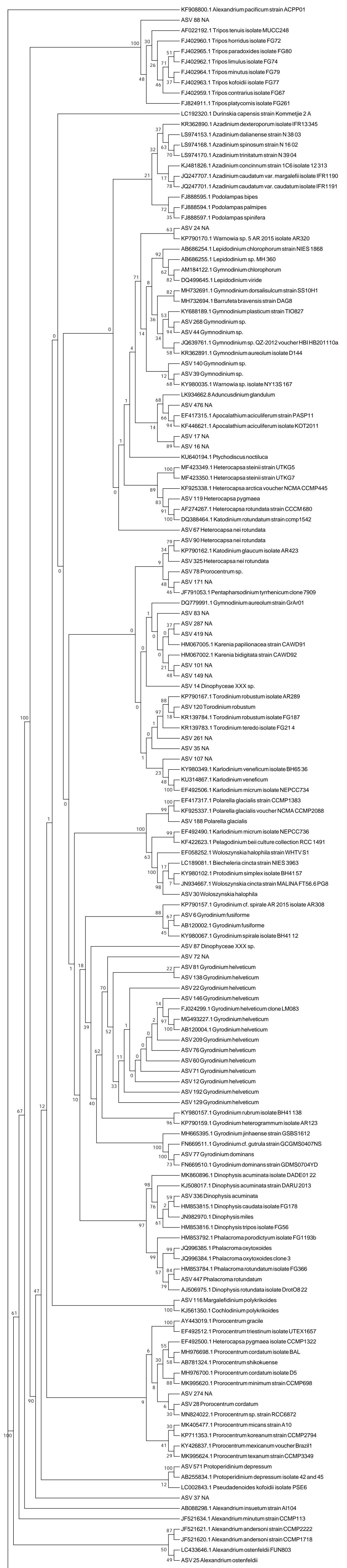

Supplement: Supplementary file 7 [file Data_Sheet_4.PDF]

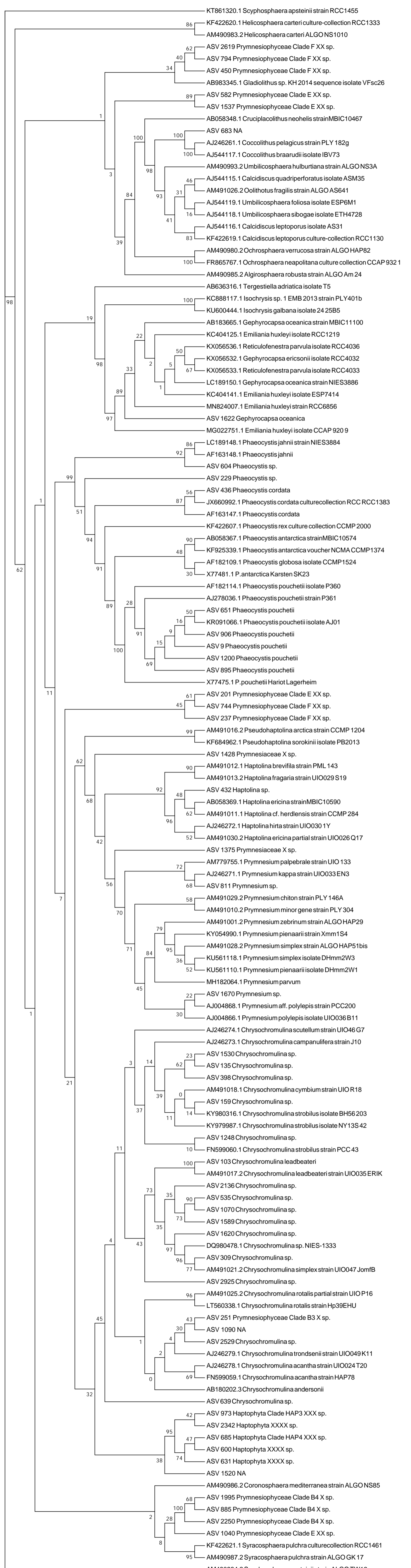

Supplement: Supplementary file 8 [file Data_Sheet_5.PDF]
